# Functional Analysis of Macromolecular Polysaccharides: Whitening, Moisturizing, Anti-Oxidant, and Cell Proliferation

**DOI:** 10.3390/antiox8110533

**Published:** 2019-11-07

**Authors:** Chien-Jen Kao, Hsin-Yu Chou, Yu-Chen Lin, Qinghong Liu, Hui-Min David Wang

**Affiliations:** 1 Department of Internal medicine of Gangshan Branch of Kaohsiung Armed Forces General Hospital, National Defense Medical Center, Kaohsiung 82049, Taiwan; kaochienjen@yahoo.com.tw; 2 Program in Tissue Engineering and Regenerative Medicine, National Chung Hsing University, Taichung 402, Taiwan; s9412105@gmail.com; 3 Graduate institute of biomedical engineering, Chung Hsing University, Taichung 402, Taiwan; 114eric0110@gmail.com; 4 Department of Chemical Engineering, National Chung Hsing University, Taichung 402, Taiwan; 5 Department of Vegetable, College of Horticulture, China Agricultural University, Beijing 100193, China; 6 Graduate Institute of Medicine, College of Medicine, Kaohsiung Medical University, Kaohsiung 807, Taiwan; 7 Department of Medical Laboratory Science and Biotechnology, China Medical University, Taichung 404, Taiwan

**Keywords:** *Achatina fulica*, *Heimiella retispora*, reactive oxygen species (ROS), collagen, moisturizing

## Abstract

In this research we utilized extracts from two different nature products, *Achatina fulica* and *Heimiella retispora*, to enhance skin moisturizing abilities, anti-oxidative properties, and cell proliferations. It was observed that two polysaccharides with anti-oxidative effects by chelating metal ions reduced oxidative stress and further blocked the formation of reactive oxygen species syntheses. To detect whether there was a similar effect within the cellular mechanism, a flow cytometry was applied for sensing the oxidative level and it was found that both materials inhibited the endogenous oxidative stress, which was induced by phorbol-12-myristate-13-acetate (PMA). Both polysaccharides also stimulated the production of collagen to maintain skin tightness and a moisturizing effect. In summary, we developed two macromolecular polysaccharides with potential applications in dermal care.

## 1. Introduction

The skin is the body’s largest organ. It consists of three layers: The epidermis, dermis, and the subcutaneous tissue. The outer layer of the epidermis consists of dead skin cells, natural oils, and lipids to protect the skin from irritants and toxins and to prevent the loss of water, biomolecules, and electrolytes [1]. When the outer layer of the epidermis is exposed to some detergent and detergent ingredients, these protective elements on the surface of the skin are peeled off. Once these irritants penetrate the outer layers of the skin, they can cause dry skin and skin health problems. One of the most important functions of the skin is to provide a barrier to prevent excessive transepidermal water loss [2,3]. Constant water movement on the skin plays an important role in the epidermal repair process. Thus, skin moisturization is very important [4]. The base layer of the epidermis is attached to the dermis, wherein the basal layer contains melanocytes. The synthesis of melanin causes the skin, eyes, and hair to darken, which is composed of colored biopolymers in the melanosomes of melanocytes. Melanin is a protective mechanism against UV damage [5,6]. A copper-containing enzyme plays a key role in melanin production. Known as tyrosinase, tyrosinase catalyzes the hydroxylation of L-tyrosine to L-3,4-dihydroxyphenylalanine (L-DOPA) and then oxidizes L-DOPA to dopaquinone [7]. Several tyrosinase inhibitors are currently used as agents for epidermal hyperpigmentation. Many skin lightening agents have been developed, such as hydroquinone, kjoic acid, and amla fruit extract powder. Ultraviolet radiation (UVR) can cause light irritation, photoaging, and carcinogenesis to induce skin inflammation. Enhanced endogenous protective mechanisms of oxidative damage are promising strategies for reducing skin damage [5,7,8,9,10,11]. Skin cells are formulated with antioxidants to eliminate ROS to maintain a balance of pro-oxidant/antioxidants. However, the proliferation of reactive oxygen species (ROS) leads to the consumption of antioxidants and the further formation of reaction products, leading to oxidative stresses [12,13]. When the skin is physically damaged, a wound is created, and the wound is repaired by inflammation, cell proliferation, and tissue remodeling. This study focuses on cell proliferation because cell proliferation is very important in the speed of wound repair.

The study of polysaccharides has been very popular in recent years. Depending on the corresponding chemical structure, polysaccharides and their derivatives have some special biological characteristics, such as biological response modifiers, anti-inflammatory, hypolipidemic, and anticoagulant. However, no one has specifically applied it to skin maintenance. In this century, snails have become available through aquaculture. Due to its traditional sensory qualities and particularly high nutritional value in Europe, especially in France, Spain, the Netherlands, Belgium, and Portugal, the use of snail-related products and the snails themselves are still considered to be extravagant [14]. The main species of edible snails belong to two families: *Helicidae* and *Achantinide*. The *Helicidae* is mainly found in Europaen countries, and *Achantinide* is usually found in African and Asian countries [15,16,17]. In the Taiwan market *Achatina fulica* exists in a traditional dish that is delicious and nutritious, called hot-fried snails. The research on *A. fulica* in the scientific field is limited, and the main research is biology [14]. Snails are rich in beneficial ingredients, the polysaccharide derivative isolated from *A. fulica* can selectively block angiogenesis in an inflammatory model induced by VEGF (vascular endothelial growth factor), and it is speculated the bioactive polysaccharide may have some health promoting activity [14]. A rot fungus is called “*Heimiella retispora”* by the Chinese, and it has been used to improve human health and longevity in the past millennium [18]. Many studies have confirmed polysaccharides isolated from *H. retispora* possess many special characteristics, such as improving insulin sensitivity and having anti-inflammatory, immunomodulatory anti-inflammatory, and anti-tumor properties [18,19,20,21]. *H. retispora* have flourished in the food and pharmaceutical industries in recent years. However, data on skin maintenance and repair of *H. retispora* is limited.

Polysaccharides have been researched and found to have many beneficial activities, but research on polysaccharides in skin care is limited. This research performed a series of biofunctional tests for two polysaccharides enriched extracts—*A. fulica* extracts and *H. retispora* extracts. Due to its moisturizing and skin-repairing properties, we discovered these two extracts have potential for the application in skin care products.

## 2. Materials and Methods

### 2.1. Chemicals and Reagents

Ascorbic acid (vitamin C), 3-(4,5-dimethylthiazol-2-yl)-2,5-diphenyltetrazolium bromide (MTT), L-3,4-dihydroxyphenylalanine (L-DOPA), dimethyl sulfoxide (DMSO), 1,1-diphenyl-2-picrylhydrazyl (DPPH), ethanol, ethylenediaminetetraacetic acid (EDTA), ferrous chloride (FeCl_2_·4H_2_O), ferric chloride (FeCl_3_), kojic acid, methanol, potassium ferricyanide (K_3_Fe(CN)_6_), 3-tert-butyl-4-hydroxyanisole (BHA), 5-hydroxy-2-hydroxymethyl-4-pyrone (kojic acid), 12-O-Tetradecanoylphorbol-13-acetate (PMA), and L-tyrosine were purchased from Sigma-Aldrich Company (St. Louis, MO, USA). Dulbecco’s modified Eagle’s medium (DMEM) and fetal bovine serum (FBS) were obtained from Gibco BRL (Gaithersburg, MD, USA). Other chemical buffers and reagents were purchased at the highest available purity and quality.

### 2.2. Extraction from Giant African Land Snail (Achatina Fulica) and Heimiella Retispora

Giant African land snails were purchased from a local snail farm, and the fruiting bodies of *H. retispora* were from the mushroom market in Kunming, Yunnan Province in China. The snails were put into a box with a sieve at the bottom, and the box was vibrated by an ultrasonic wave instrument to stimulate the snails’ secretions. The secretions were collected and diluted with reverse osmosis (RO) water 1000 times. Ammonium sulfate was added into the snail secreted solution until 80% saturation was reached. After standing at 4 °C for 12 h, the sediments were collected by centrifugation at 40 *g* × 30 min, at 4 °C. The sediments were dialyzed with molecular weight cut-off (MWCO) of 10 kDa against water. After dialyzing, the solution was centrifuged at 300 *g* × 30 min, at 4 °C to discharge the sediments and collected the supernatant. After the supernatant was lyophilized, the snail secretion powder was stored at −20 °C.

The fruiting bodies of *H. retispora* (500 g) were homogenized with a waring blender. The homogenized sample was heated in a water boiler at 90 °C for 4 h. The supernatant were collected by a centrifugation at 300 *g* × 30 min, at 4 °C. Methanol was added to the supernatant until the concentration reached 70% (*v/v*). After standing at room temperature for 10 h, the sediment was collected by a centrifugation at 500 *g* × 30 min, at 4 °C. The sediments were dialyzed with MWCO 35 kDa against water. After dialyzing, the solution was centrifuged at 300 *g* × 30 min, at 4 °C to discharge the sediments and to collect the supernatant. After the supernatant were lyophilized, the snail secretion powder was stored at −20 °C.

### 2.3. Evaporation Rate of A. fulica Extracts and H. retispora Extracts

A simple method was used to estimate the evaporation rate of *A. fulica* extracts and *H. retispora* extracts. Samples mixed with 1 mL water were applied to a 4-cm in diameter glass dish. This amount was chosen so the sample fluid covered the entire filter glass at all times during the experiment. The dish was placed on a scale in a draft-free environment at 32 °C for 2 h (the skin surface is typically about 32 °C). The weight of the samples was recorded at time zero to determine the exact amount applied and two hours later to determine the amount that had evaporated during that period, and the evaporation rate was computed as:

(1) $$\mathrm{Evaporation}\;\mathrm{rate}\;(\%)\;=\;\frac{\left(\Delta M_{sample}\right)}{\left(\Delta M_{control}\right)}\;\times \;100\%$$

where Δ*M_sample_* means weight change of sample; Δ*M_control_* means weight change of control.

### 2.4. Determination of 1,1-Diphenyl-2-Picrylhydrazyl (DPPH) Radical Scavenging Capacity

DPPH has stable free radicals and it is an anti-oxidant assay to detect the ability of anti-oxidants to scavenge free radicals [22]. It is a purple reagent, which transforms into yellow if the hydrogen of DPPH transfers to anti-oxidants. Correction concentration samples were added to DPPH (60 μM), and the DPPH became a bright color at 517 nm because the optical absorbance reduced. The percentages of remaining DPPH and the sample were used to calculate the amount of anti-oxidant required. Scavenging activity (%) was calculated according to:

(2) $$\mathrm{Scavenging}\;\mathrm{activity}\;(\%)\;=\;\frac{\left(A_{sample}-A_{blank}\right)}{A_{control}}\;\times \;100\%$$

where *A_sample_* means absorption of samples at 595 nm wavelength; *A_blank_* means absorption of blank at 595 nm wavelength; *A_control_* means absorption of control at 595 nm wavelength.

### 2.5. Metal Chelating Activity

Metal ion can cause lipid peroxidation, especially ferrous ion which is pro-oxidant. The samples were filled into 10 μL FeCl_2_·4H_2_O (2 mM) and then mixed in 20 μL ferrozine (5 mM). The admixture was shaken and held at 25 °C for 10 min. The absorbance of the sample solution was observed at 562 nm. EDTA acted as a positive control, and the chelating power calculation formula was based on Equation (2).

### 2.6. Reducing Power

The determination of the reduction force is based on the method of Oyaizu. Briefly, samples were mixed with phosphate-buffered saline buffer (85 μL, 67 mM, pH6.8) and potassium ferricyanide (K_3_Fe(CN)_6_) (2.5 μL, 20%). Then, the reaction was carried out at 50 °C for 20 min. Following this, 160 μL of trichloro acetic acid (10%) was mixed with the reaction and 20 min was spent to centrifuge 300×. The optical density was determined at 700 nm through a 96-well plate after the solution was mixed with 2% FeCl3 (25 μL). Our positive control was based on butylated hydroxyanisole (BHA), and a higher reducing performance had the property of higher light absorption. 

### 2.7. Cytotoxicity Examinations

A total of 5% CO_2_ at 37 °C was used to cultivate human dermal fibroblasts cell line HS68 (ATCC^®^ CRL-1635™) (ATCC, Manassas, VA, USA) cell at a consistent monolayer culture of Dulbecco’s modified Eagle medium (DMEM) for 24 h. Fetal bovine serum (FBS) (10%), penicillin (100 U/mL), streptomycin (100 mg/mL), amphotericin B (0.25 µg/mL), and amphotericin B (0.25 μg/mL) were the ingredients of DMEM. *A. fulica* mucus were dissolved in adding dimethyl sulfoxide (DMSO) at different concentrations without any impurity, and the DMSO concentration was less than 1.0% compared to the final working volume. The influences of testing samples on cell development were estimated with 3-(4,5-dimethylthiazol-2-yl)-2,5-diphenyltetrazolium bromide (MTT) assay. Cells were seeded at 1 × 10^4^ cells/well in 96-well plates and allowed to hatch for 24 h before adding the extracts. After 24 h, the MTT solution was dispensed into each well. After another two hours, the culture medium was discarded, and DMSO was added to each well. The absorbance of the formazan salt was 595 nm, and the cell viability was computed as Equation (3).

(3) $$\mathrm{Cell}\;\mathrm{viability}\;(\%)\;=\;\frac{\left(A_{sample}-A_{blank}\right)}{\left(A_{control}-A_{blank}\right)}\;\times \;100\%$$

### 2.8. B16-F10 Cellular Tyrosinase Activity

According to the previous assay, the tyrosinase activity was turned on the dopachrome formation rate [23]. Melanoma B16-F10 cells (10^5^ cells/well) were added to 1000 μL of medium and seeded in a 12-well plate. During the next 24 h, they were treated by assigned concentrations of graphene oxide nanoribbons. B16-F10 cells were lysed with 1% Triton X-100/ phosphate buffered saline (PBS) buffer after PBS washing, and then, 50 μL of 2 mM L-tyrosine was added. The mixture was incubated in darkness for 3 h at 37 °C. The optical absorbance was spectrophotometrically monitored at 490 nm. The tyrosinase activity evaluation formula was similar to Equation (2).

### 2.9. Detection of ROS by 2’,7’-dichlorodihydrofluorescein Diacetate (DCFDA) Stain

HS68 cells were seeded in a 6-well micro-plate at a density of 1.2 × 10^5^ cells/well as described [24]. After 24 h, the cells were treated with 10 and 25 mg/L of samples. Cells were then washed twice with PBS buffer, suspended with 1 mL trypsin, and loaded with DCFDA (5 μM) for 30 min at 37 °C in DMEM without phenol red. Individual cells were suspended by gentle pipetting up and down three times prior to flow cytometry analyses. DCFDA was illuminated with a 488 nm laser and detected at 535 nm.

### 2.10. Statistical Analysis

Three of each concentration for the standard and the samples were used. Using Student’s *t*-test, the results were statistically compared and were expressed using the average of the mean values ± standard deviation (SD).

## 3. Results and Discussion

### 3.1. Moisturizing Activities

**Figure 1:**
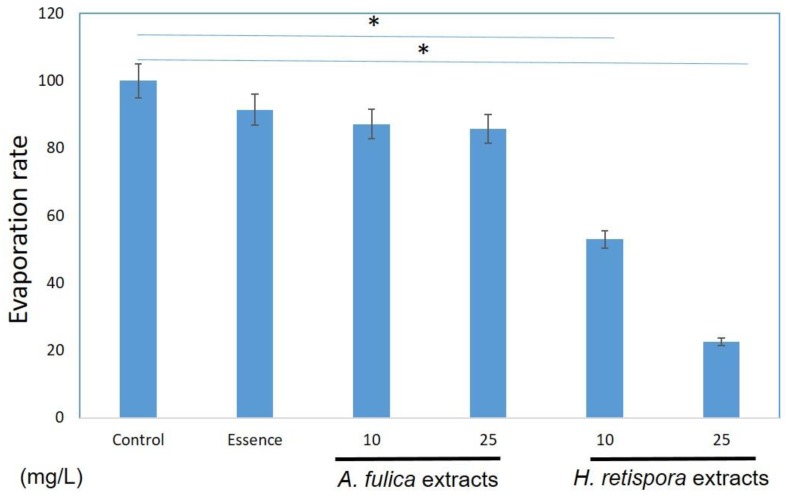
*Achatina fulica* extracts and *Heimiella retispora* extracts showed potential moisturizing activities. (Data represents mean ± S.D of three independent experiments performed. * *p* < 0.01).

The moisture of the skin is strongly correlated with skin maintenance. The moist environment can decrease the rate of skin aging, promote wound repair, and reduce scar production. Therefore, excellent moisturizing ability is very important for the application of the material in the skin. In the article, we used *A. fulica* extracts and *H. retispora* extracts to measure the moisturizing activities of the skin. We found *A. fulica* extracts decreased the evaporation rates by 12.8% and 14.3% at a concentration of 10 mg/mL and 25mg/mL, and the *H. retispora* extracts had a better effect on moisturizing function; it decreased 47.1% and 77.5% evaporation at a concentration of 10 mg/L and 25 mg/L. Moreover, marketed essence has only 8% moisturizing power (Figure 1). Some previous reports explored the use of natural products in moisturizing research, but most of them found indirect evidence, including the production of hyaluronic acid and collagen in skin cells [25,26]. However, the outermost layer of the skin is the stratum corneum rather than the cells, and the water does not directly evaporate from the cells. Therefore, we made a measurement of moisture retention, making sure that it reduced water evapotranspiration. From the results of Figure 1, both *A. fulica* extracts and *H. retispora* extracts have the effects of inhibiting the evaporation of water directly, and the potential to be utilized in skin moisturizing.

### 3.2. Anti-Oxidative Properties of the A. fulica Extracts and H. retispora Extracts

Antioxidant properties are much more abundant in the reporting of natural substances than in moisturizing [22,27], and the use of antioxidant properties has a good effect in areas such as inflammation and whitening. In this research, we used multiple methods to determine these two natural substances, including DPPH, chucking, reducing power tests intracellular oxidative stress analysis. We found these extracts were particularly effective on the chelating test.

#### 3.2.1. *A. fulica* Extracts and *H. retispora* Extracts Had No DPPH Free Radical Scavenging Activity

DPPH free radical scavenging activity is a common antioxidant activity method. However, the two extracts had no significant effect in the DPPH test. We speculate *A. fulica* extracts and *H. retispora* extracts do not work in DPPH. Since the material properties may vary, different antioxidant detection methods will have different reactions (Table 1).

#### 3.2.2. Ferrous Ions Chelating Capacity Measurements

In the Ferrous ions chelating capacity measurements, ferrozine reacts with iron ions and turns into a dark red color. Once the analyte can be chelated with iron ions, which causes a reduction reaction, the Fe^2+^ complex is destroyed and the color is lightened. EDTA was used as the positive control at a concentration of 100 μM. In Table 1, *A. fulica* extracts and *H. retispora* extracts had chelating properties at 25 mg/L (40.15 ± 0.05%, 28.03 ± 0.04%), while the EDTA reached the same condition at 100 μM (87.38 ± 0.08%). It can be observed from the experimental results that *A. fulica* extracts and *H. retispora* extracts tend to achieve antioxidant effects by means of chelating iron ions.

#### 3.2.3. Ferric Reducing Antioxidant Power (FRAP) Index Assessments

This study quantifies the Fe (III)-ferricyanide complex. The reduction reaction will change the complex from yellow to blue. Table 1 shows the reducing powers of *A. fulica* extracts and *H. retispora* extracts were OD 0.25 and 0.17 at 10 mg/L. From three different aspects of antioxidant response testing, we can observe *A. fulica* and *H. retispora* extracts have antioxidant properties in vitro. We further test the intracellular antioxidant assay to examine the antioxidant ability in the skin cells. 

#### 3.2.4. *A. fulica* Extracts and *H. retispora* Extracts Inhibit Intracellular ROS Accumulation

**Figure 2:**
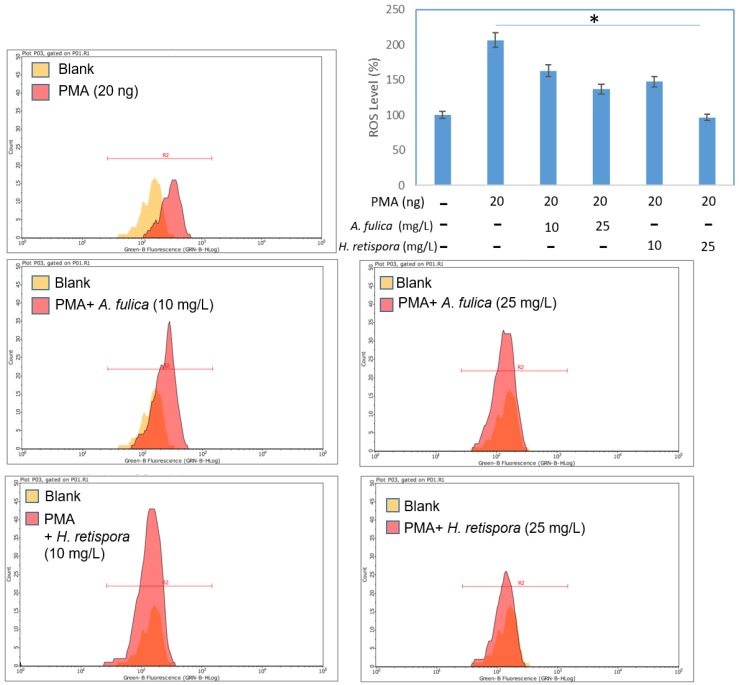
The 2’,7’-dichlorodihydrofluorescein diacetate (DCFDA) assay results showing that *A. fulica* extracts and *H. retispora* extracts treatment decreased ROS production in HS68 cells. The phorbol-12-myristate-13-acetate (PMA) was used as negative control to increase the oxidative level. Data represents mean ± S.D of three independent experiments performed. (Data represents mean ± S.D of three independent experiments performed. * *p* < 0.01).

Previous reports revealed ROS caused damage on cellular biological morphological structures, including cell membranes, organelles, DNA, and protein configurations [28,29,30]. Meanwhile, a high level oxidative stress induced massive productions of melanin. Therefore, reducing oxidative stress is one good guideline to decrease melanin production. We used a cell-permeant DCFDA stain assay to test whether *A. fulica* extracts and *H. retispora* extracts treatments diminished intracellular ROS levels. Chemical fluorescent DCFDA staining is often applied to measure oxidative stress, which can be defined as the absence of oxidation. Typically, DCFDA is introduced into target cells via a small amount of aqueous solution, and then rapidly diffuses through the cell membrane as a colorless probe. Once the two acetate groups are cleaved by esterases within the cell, DCFDA fluorescence is detectable. A valuable property of DCFDA is that it cannot exit the cell while it has been cleaved within the cell. This increasing period of time during DCFDA reactions can be determined as an oxidative stress indicator. We used phorbol 12-myristate 13-acetate (PMA, 20 ng/mL), which is an inducer for endogenous superoxide production, as a negative control to induce oxidative stress, and then treated *A. fulica* extracts and *H. retispora* extracts for 24 h [31]. When the solution was comprised of 20 ng/mL of PMA, it enhanced the relative expressions of ROS by 106%, and adding PMA enlarged the ROS expression while AFMPS and GLNPS lessened the relative expression. There was a parallel trend among *A. fulica* extracts and *H. retispora* extracts with PMA, with the latter of the two displaying a larger difference compared to the solution containing only PMA. We observed both of these two materials decreased ROS levels, and the anti-oxidative effect of *H. retispora* was better than *A. fulica* (Figure 2). It showed similar results on in vitro antioxidant tests in Table 1.

### 3.3. Cell Growth of A. fulica Extracts and H. retispora Extracts Treated in Human Fibroblasts

**Figure 3:**
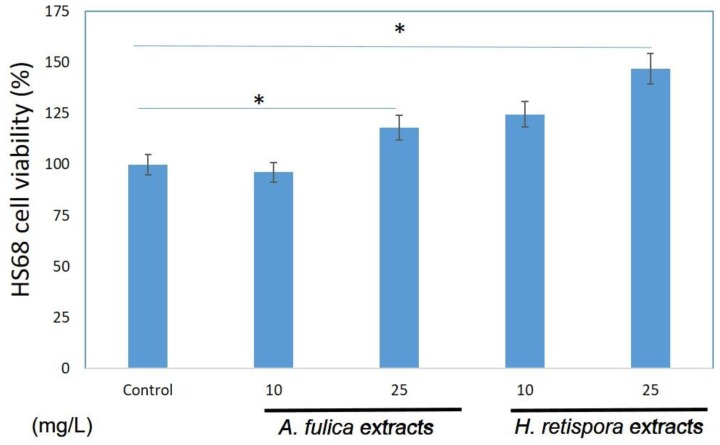
*A. fulica* extracts and *H. retispora* extracts effects on human cell viability with various doses. Fibroblasts were seeded in a 96-well micro titer plate which had a density of about 1 × 10^4^ cells/well and treated with 1, 5, and 10 mg/L of *A. fulica* extracts and *H. retispora* extracts for 24 h. The cell viability of fibroblasts was measured by MTT assay 24 h after compound treatment. (Data represents mean ± S.D of three independent experiments performed. * *p* < 0.01).

Figure 3 shows the *A. fulica* extracts and *H. retispora* extracts increased the cell growth rate. The *A. fulica* extracts increased the growth rate by 118% 24 h after the addition of the extract, while *H. retispora* extracts increased by 146% at a concentration 25 mg/L. The chart shows as the length of concentration after the addition of *A. fulica* extracts and *H. retispora* extracts into the medium increased, the cell viability of fibroblasts was enhanced as well. In a previous study, both *A. fulica* extracts and *H. retispora* extracts were found to contain polysaccharides [14], Polysaccharides have the effect of promoting growth and can be applied to cell repair, skin care, and scar removal. This has also been verified in this experiment, especially in GLNPS with better promotion of proliferation.

### 3.4. Collagen Productions in Sirius Red Assays

**Figure 4:**
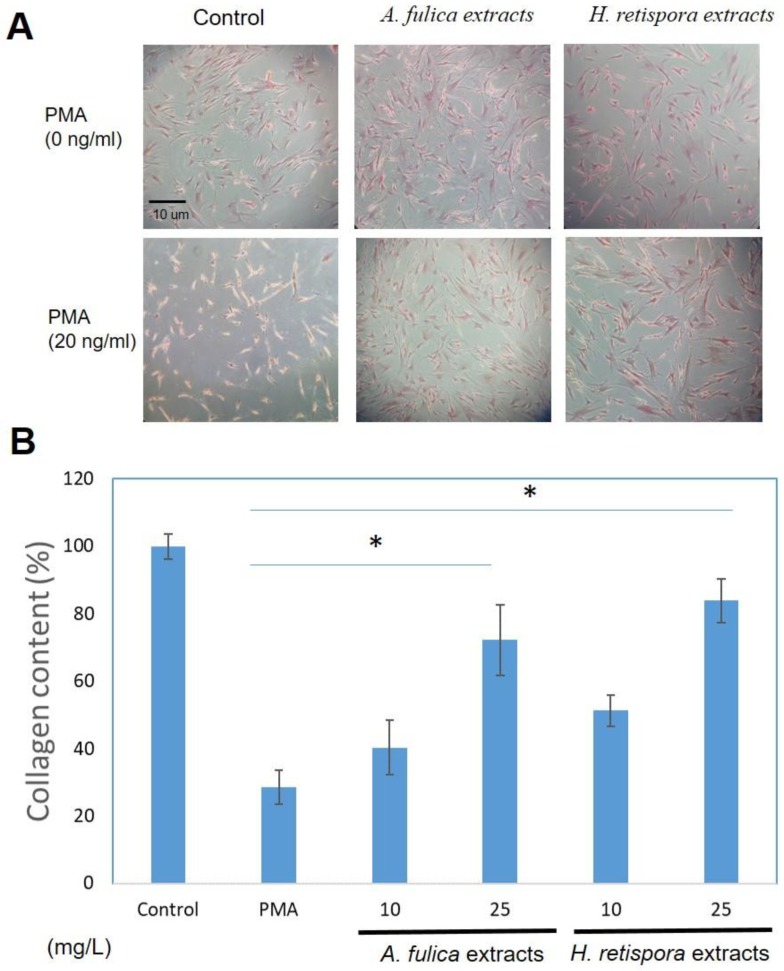
**(A)** HS68 cells collagen production with *A. fulica* extracts and *H. retispora* extracts treatments in Sirius red assay. PMA is used as negative control. (**B**) The quantitative data of collagen production; Data represents mean ± S.D of three independent experiments performed. (Data represents mean ± S.D of three independent experiments performed. * *p* < 0.01).

The increased oxidative stress not only destroys the inside of the cell, but also affects the extracellular matrix. The change in the extracellular matrix is also one of the reasons for inducing cell carcinogenesis and tumor metastasis [32,33]. In previous experiments, we observed *A. fulica* extracts and *H. retispora* extracts have antioxidant properties, so we did a test of collagen in the cell content. PMA was used as a negative control, which induced oxidative stress and inhibited the production of collagen in the cells. After adding *A. fulica* extracts and *H. retispora* extracts, we observed a significant response of collagen, which was restored at a concentration of 25 mg/L. 44.9% and 55.4% (Figure 4), meaning *A. fulica* extracts and *H. retispora* extracts contribute to the repair of extracellular matrices.

### 3.5. A. fulica Extracts and H. retispora Extracts on B16-F10 Cellular Tyrosinase Activity

**Figure 5:**
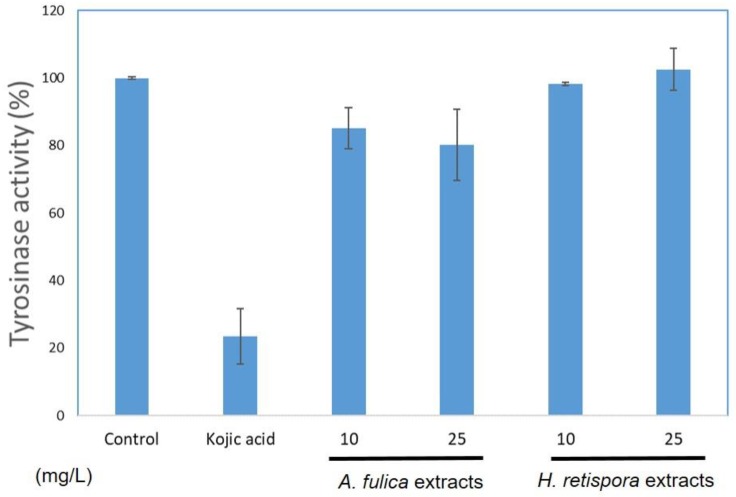
The inhibitory effects of tyrosinase activity. Treated with 10 and 25 μg/mL of *A. fulica* extracts and *H. retispora* extracts.

Tyrosinase, a rate limiting enzyme, plays a critical role in pigment biosynthesis reactions. Other downstream enzymes influence the differences in color types for the syntheses of eumelanin and phenomelanin. To observe whether the four nanocarbons reduced melanin synthesis by down-regulating tyrosinase, in vitro tyrosinase activity was examined in the melanoma cell B16-F10 type. The data showed *A. fulica* extracts and *H. retispora* extracts inhibited 17.1 ± 2.9% and 12.6 ± 3.5% of the tyrosinase activity at a concentration of 25 mg/L, with both of them being in a dose-dependent manner from 10–25 mg/L. We observed *A. fulica* extracts and *H. retispora* extracts had better properties to suppress tyrosinase activity (Figure 5). Melanin is the source of skin color, which absorbs UV radiation to avoid UV-induced DNA damage and mutation. Although melanin can protect the skin from UV damage, the over expression of melanin also causes some skin disorders [34]. The experimental results suggested *A. fulica* extracts and *H. retispora* extracts could inhibit the synthesis of melanin. Table 1 shows the effect of chelating metal ion *A. fulica* extracts is better than the *H. retispora* extracts, meaning *A. fulica* extracts could be more positively used in cosmetic production as whitening agents than *H. retispora* extracts.

## 4. Conclusions

To sum up, we tested *A. fulica* and *H. retispora* macromolecular polysaccharide extracts as potential raw materials for cosmeceutical applications because of the multiple biofunctional properties. The experiments showed that these two extracts played roles as antioxidant ingredients and electron donors to stop free radical chain reactions. At the same time, *A. fulica* extracts inhibited tyrosinase activity, and *H. retispora* extracts promoted cell proliferation.

## Figures and Tables

**Table 1:** Antioxidant activities of *A. fulica* extracts and *H. retispora* extracts, including reducing power, 1,1-diphenyl-2-picrylhydrazyl (DPPH) free radical scavenging activity, and ferrous ion chelating power. Data represents mean ± S.D of three independent experiments performed.

Samples	Concentration (mg/L)	DPPH (%)	Chelating (%)	Reducing Power (OD700)
Vitamin C	100 μM	87.4 ± 0.1	-	-
EDTA	100 μM	-	85.6 ± 0.1	-
BHA	100 μM	-	-	0.67 ± 0.02
*A. fulica* extracts	10	21.83 ± 0.06	N/A	0.25 ± 0.06
	25	40.15 ± 0.03	N/A	0.24 ± 0.02
*H. retispora* extracts	10	14.48 ± 0.05	N/A	0.17 ± 0.01
	25	28.03 ± 0.04	N/A	0.18 ± 0.01

N/A: Unable to measure valid values.

## Data Availability

The data used to support the findings of this study are available from the corresponding author upon request.
